# 9-Ethyl-*N*-(3-nitro­benzyl­idene)-9*H*-carbazol-3-amine

**DOI:** 10.1107/S1600536811023890

**Published:** 2011-06-25

**Authors:** R. Archana, E. Yamuna, K. J. Rajendra Prasad, A. Thiruvalluvar, R. J. Butcher

**Affiliations:** a PG Research Department of Physics, Rajah Serfoji Government College (Autonomous), Thanjavur 613 005, Tamilnadu, India; b Department of Chemistry, Bharathiar University, Coimbatore 641 046, Tamilnadu, India; c Department of Chemistry, Howard University, 525 College Street NW, Washington, DC 20059, USA

## Abstract

The title compound, C_21_H_17_N_3_O_2_, crystallizes with two mol­ecules in the asymmetric unit. The carbazole groups show relatively small deviations from planarity [maximum displacements from the mean carbazole plane are 0.077 (7) and 0.101 (7) Å]. The dihedral angles between the 3-nitro­benzyl­idene­amine and carbazole groups are 37.9 (1) and 37.0 (1)° in the two mol­ecules.

## Related literature

For anti-convulsant and diuretic activity of rimcazole (systematic name 9-{3-[(3*R*,5*S*)-3,5-dimethyl­piperazin-1-yl]prop­yl}-9*H*-carbazole) and other *N*-alkyl­amino carbazoles, see: Ferris *et al.* (1986 ▶), Shoeb *et al.* (1973 ▶). For a related structure, see: Archana *et al.* (2010 ▶).
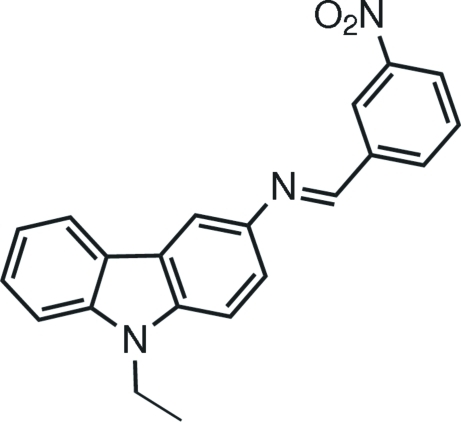


## Experimental

### 

#### Crystal data

C_21_H_17_N_3_O_2_
*M* *_r_* = 343.38
Monoclinic, 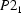

*a* = 8.1550 (4) Å
*b* = 13.5093 (7) Å
*c* = 16.0248 (9) Å
β = 103.899 (5)°
*V* = 1713.74 (16) Å^3^
*Z* = 4
Cu *K*α radiation
μ = 0.71 mm^−1^
*T* = 295 K
0.44 × 0.41 × 0.35 mm

#### Data collection

Oxford Diffraction Xcalibur Ruby Gemini diffractometer
Absorption correction: multi-scan (*CrysAlis PRO*; Oxford Diffraction, 2010 ▶) *T*_min_ = 0.830, *T*_max_ = 1.000
6983 measured reflections
3509 independent reflections
2458 reflections with *I* > 2σ(*I*)
*R*_int_ = 0.034

#### Refinement

*R*[*F* ^2^ > 2σ(*F* ^2^)] = 0.080
*wR*(*F* ^2^) = 0.246
*S* = 1.08
3509 reflections
471 parameters
1 restraint
H-atom parameters constrained
Δρ_max_ = 0.32 e Å^−3^
Δρ_min_ = −0.29 e Å^−3^

### 

Data collection: *CrysAlis PRO* (Oxford Diffraction, 2010 ▶); cell refinement: *CrysAlis PRO*; data reduction: *CrysAlis PRO*; program(s) used to solve structure: *SHELXS97* (Sheldrick, 2008 ▶); program(s) used to refine structure: *SHELXL97* (Sheldrick, 2008 ▶); molecular graphics: *PLATON* (Spek, 2009 ▶); software used to prepare material for publication: *PLATON*.

## Supplementary Material

Crystal structure: contains datablock(s) global, I. DOI: 10.1107/S1600536811023890/ya2140sup1.cif

Structure factors: contains datablock(s) I. DOI: 10.1107/S1600536811023890/ya2140Isup2.hkl

Supplementary material file. DOI: 10.1107/S1600536811023890/ya2140Isup3.cml

Additional supplementary materials:  crystallographic information; 3D view; checkCIF report

## Figures and Tables

**Fig. 1.**
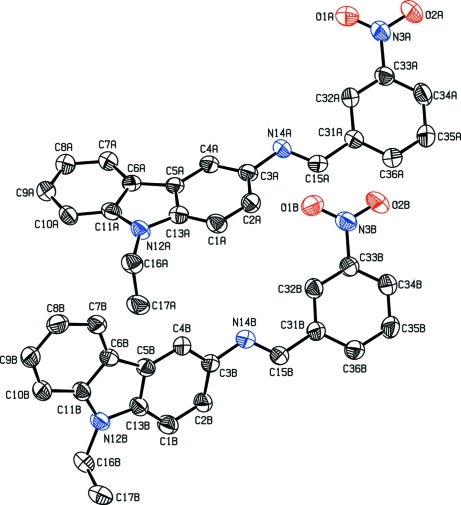
Asymmetric unit of the title compound; displacement ellipsoids are drawn at the 30% probability level. H atoms were omitted for clarity.

## supplementary crystallographic information

### Comment

*N*-Alkylamino carbazoles show significant anti-convulsant and diuretic activity (Shoeb *et al.*, 1973). One of them, rimcazole is a well known anti-pyretic and neuroleptic agent (Ferris *et al.*, 1986). It was found to be a specific competitive antagonist of s-sites in the brain; it reverses psychotic conditions induced in humans by phencyclidine and/or s-opiod antagonists, probably by binding to receptors in the brain.

Archana *et al.* (2010) have reported a crystal structure of substituted carbazole derivative, in which the carbazole unit is almost planar. The title compound, C_21_H_17_N_3_O_2_, crystallizes with two molecules (A and B) in the asymmetric unit (Fig. 1). The carbazole groups show relatively small deviations from planarity [maximum displacement of the C9A atom from the mean carbazole plane in molecule A is equal to 0.077 (7) Å, displacement of the C9B atom in B is 0.101 (7) Å]. The dihedral angles between the 3-nitro-benzylideneamine group and the carbazole group are 37.9 (1)° and 37.0 (1)° in A and B respectively.

### Experimental

Herein, we report a simple and effective method of high-yield synthesis of the title compound. A mixture of *m*-nitrobenzaldehyde (0.154 g, 0.001 mol), 3-amino-9-ethylcarbazole (0.210 g, 0.001 mol) and sodium bicarbonate (0.080 g, 0.001 mol) was ground and kept at room temperature for 2 h. The reaction was monitored by TLC. Then the solid was quenched with crushed ice under stirring and acidified with dilute hydrochloric acid till the solution became acidic (litmus color change from blue to red). The product was extracted with ethylacetate and dried over anhydrous sodium sulfate. Upon removal of the solvent, a yellow crude product was obtained. It was purified by column chromatography over silica gel using petroleum ether: ethylacetate (95:5, *v*/*v*) mixture as eluent to afford the title compound (yield: 0.274 g, 80%); the pale-orange product was recrystallized from ethanol.

### Refinement

As there are no significant anomalous scatterers in the molecule, the Friedel pairs were merged. H atoms were positioned geometrically and allowed to ride on their parent atoms, with C—H = 0.93, 0.96 and 0.97 Å for C*sp*^2^, methyl and methylene H atoms, respectively. *U*_iso_(H) = *xU*_eq_(C), where *x* = 1.5 for methyl H atoms and 1.2 for other C-bound H atoms.

### Crystal data

**Table:**

C_21_H_17_N_3_O_2_	*F*(000) = 720
*M**_r_* = 343.38	*D*_x_ = 1.331 Mg m^−^^3^
Monoclinic, *P*2_1_	Melting point: 352 K
Hall symbol: P 2yb	Cu *K*α radiation, λ = 1.54184 Å
*a* = 8.1550 (4) Å	Cell parameters from 2308 reflections
*b* = 13.5093 (7) Å	θ = 5.6–73.4°
*c* = 16.0248 (9) Å	µ = 0.71 mm^−^^1^
β = 103.899 (5)°	*T* = 295 K
*V* = 1713.74 (16) Å^3^	Block, pale-orange
*Z* = 4	0.44 × 0.41 × 0.35 mm

### Data collection

**Table:**

Oxford Diffraction Xcalibur Ruby Gemini diffractometer	3509 independent reflections
Radiation source: Enhance (Cu) X-ray Source	2458 reflections with *I* > 2σ(*I*)
graphite	*R*_int_ = 0.034
Detector resolution: 10.5081 pixels mm^-1^	θ_max_ = 73.6°, θ_min_ = 5.6°
ω scans	*h* = −10→7
Absorption correction: multi-scan (*CrysAlis PRO*; Oxford Diffraction, 2010)	*k* = −16→9
*T*_min_ = 0.830, *T*_max_ = 1.000	*l* = −19→18
6983 measured reflections	

### Refinement

**Table:**

Refinement on *F*^2^	Primary atom site location: structure-invariant direct methods
Least-squares matrix: full	Secondary atom site location: difference Fourier map
*R*[*F*^2^ > 2σ(*F*^2^)] = 0.080	Hydrogen site location: inferred from neighbouring sites
*wR*(*F*^2^) = 0.246	H-atom parameters constrained
*S* = 1.08	*w* = 1/[σ^2^(*F*_o_^2^) + (0.1348*P*)^2^ + 0.51*P*] where *P* = (*F*_o_^2^ + 2*F*_c_^2^)/3
3509 reflections	(Δ/σ)_max_ = 0.001
471 parameters	Δρ_max_ = 0.32 e Å^−^^3^
1 restraint	Δρ_min_ = −0.29 e Å^−^^3^

### Special details

**Table:**

Geometry. Bond distances, angles *etc*. have been calculated using the rounded fractional coordinates. All su's are estimated from the variances of the (full) variance-covariance matrix. The cell e.s.d.'s are taken into account in the estimation of distances, angles and torsion angles
Refinement. Refinement of *F*^2^ against ALL reflections. The weighted *R*-factor *wR* and goodness of fit *S* are based on *F*^2^, conventional *R*-factors *R* are based on *F*, with *F* set to zero for negative *F*^2^. The threshold expression of *F*^2^ > 2σ(*F*^2^) is used only for calculating *R*-factors(gt) *etc*. and is not relevant to the choice of reflections for refinement. *R*-factors based on *F*^2^ are statistically about twice as large as those based on *F*, and *R*- factors based on ALL data will be even larger.

### Fractional atomic coordinates and isotropic or equivalent isotropic displacement parameters (Å^2^)

**Table:**

	*x*	*y*	*z*	*U*_iso_*/*U*_eq_	
O1A	−0.0282 (8)	0.1393 (7)	0.4830 (4)	0.105 (3)	
O2A	0.1202 (9)	0.1178 (9)	0.6087 (4)	0.140 (4)	
N3A	0.1053 (9)	0.1305 (6)	0.5327 (4)	0.082 (3)	
N12A	0.1520 (8)	0.1142 (5)	−0.1190 (3)	0.068 (2)	
N14A	0.2402 (7)	0.1332 (5)	0.2351 (3)	0.0687 (19)	
C1A	0.3254 (9)	0.0611 (6)	0.0235 (4)	0.072 (3)	
C2A	0.3430 (9)	0.0681 (6)	0.1102 (5)	0.068 (2)	
C3A	0.2341 (9)	0.1275 (6)	0.1455 (4)	0.066 (2)	
C4A	0.1066 (8)	0.1804 (6)	0.0897 (4)	0.064 (2)	
C5A	0.0895 (8)	0.1752 (5)	0.0019 (4)	0.059 (2)	
C6A	−0.0291 (8)	0.2176 (5)	−0.0709 (4)	0.061 (2)	
C7A	−0.1635 (9)	0.2809 (7)	−0.0821 (5)	0.074 (3)	
C8A	−0.2614 (10)	0.3017 (7)	−0.1638 (5)	0.079 (3)	
C9A	−0.2195 (10)	0.2607 (7)	−0.2351 (5)	0.079 (3)	
C10A	−0.0813 (10)	0.1994 (6)	−0.2275 (4)	0.068 (3)	
C11A	0.0148 (9)	0.1772 (6)	−0.1449 (5)	0.067 (2)	
C13A	0.1988 (8)	0.1136 (5)	−0.0308 (4)	0.063 (2)	
C15A	0.3788 (9)	0.1236 (6)	0.2892 (4)	0.069 (3)	
C16A	0.2434 (11)	0.0668 (7)	−0.1766 (5)	0.077 (3)	
C17A	0.3884 (10)	0.1283 (8)	−0.1880 (5)	0.086 (3)	
C31A	0.3893 (8)	0.1261 (6)	0.3828 (4)	0.066 (2)	
C32A	0.2451 (9)	0.1300 (6)	0.4137 (4)	0.066 (2)	
C33A	0.2603 (9)	0.1313 (6)	0.5004 (5)	0.070 (2)	
C34A	0.4152 (11)	0.1297 (7)	0.5583 (5)	0.083 (3)	
C35A	0.5585 (10)	0.1295 (8)	0.5269 (5)	0.083 (3)	
C36A	0.5457 (9)	0.1265 (7)	0.4407 (5)	0.075 (3)	
O1B	0.4490 (7)	0.8842 (5)	0.4146 (4)	0.093 (2)	
O2B	0.6022 (9)	0.8772 (6)	0.5430 (4)	0.105 (3)	
N3B	0.5850 (9)	0.8824 (5)	0.4656 (4)	0.079 (2)	
N12B	0.6177 (7)	0.9094 (5)	−0.1890 (3)	0.0683 (19)	
N14B	0.7154 (7)	0.8953 (5)	0.1660 (4)	0.068 (2)	
C1B	0.7939 (10)	0.9677 (6)	−0.0465 (5)	0.076 (3)	
C2B	0.8102 (9)	0.9611 (7)	0.0408 (4)	0.070 (3)	
C3B	0.7059 (8)	0.8992 (6)	0.0763 (4)	0.063 (2)	
C4B	0.5824 (8)	0.8446 (5)	0.0241 (4)	0.061 (2)	
C5B	0.5615 (8)	0.8491 (5)	−0.0652 (4)	0.060 (2)	
C6B	0.4411 (8)	0.8060 (6)	−0.1376 (4)	0.066 (3)	
C7B	0.3071 (9)	0.7411 (7)	−0.1470 (5)	0.072 (3)	
C8B	0.2099 (10)	0.7211 (8)	−0.2275 (5)	0.084 (3)	
C9B	0.2454 (10)	0.7646 (7)	−0.3004 (5)	0.083 (3)	
C10B	0.3792 (10)	0.8263 (7)	−0.2938 (4)	0.078 (3)	
C11B	0.4788 (9)	0.8478 (5)	−0.2124 (4)	0.063 (2)	
C13B	0.6670 (9)	0.9118 (6)	−0.0994 (4)	0.065 (2)	
C15B	0.8523 (9)	0.9092 (6)	0.2203 (4)	0.069 (3)	
C16B	0.7073 (11)	0.9593 (6)	−0.2463 (5)	0.076 (3)	
C17B	0.8592 (11)	0.9026 (8)	−0.2563 (6)	0.093 (3)	
C31B	0.8645 (9)	0.9048 (6)	0.3131 (5)	0.067 (2)	
C32B	0.7214 (9)	0.8965 (6)	0.3455 (4)	0.068 (2)	
C33B	0.7393 (9)	0.8892 (6)	0.4328 (4)	0.069 (2)	
C34B	0.8935 (10)	0.8861 (7)	0.4902 (5)	0.077 (3)	
C35B	1.0380 (10)	0.8944 (8)	0.4593 (5)	0.082 (3)	
C36B	1.0220 (9)	0.9035 (7)	0.3711 (5)	0.075 (3)	
H1A	0.39812	0.02142	0.00140	0.0854*	
H2A	0.42871	0.03277	0.14695	0.0816*	
H4A	0.03272	0.21936	0.11161	0.0771*	
H7A	−0.18962	0.31035	−0.03446	0.0883*	
H8A	−0.35475	0.34315	−0.17058	0.0944*	
H9A	−0.28628	0.27474	−0.28952	0.0952*	
H10A	−0.05286	0.17356	−0.27590	0.0819*	
H15A	0.47691	0.11483	0.27022	0.0823*	
H16A	0.16631	0.05586	−0.23212	0.0923*	
H16B	0.28490	0.00285	−0.15317	0.0923*	
H17A	0.34801	0.19211	−0.21020	0.1293*	
H17B	0.44259	0.09613	−0.22762	0.1293*	
H17C	0.46804	0.13625	−0.13358	0.1293*	
H32A	0.13894	0.13171	0.37583	0.0799*	
H34A	0.42308	0.12868	0.61717	0.0991*	
H35A	0.66446	0.13148	0.56485	0.1003*	
H36A	0.64332	0.12469	0.42032	0.0896*	
H1B	0.86486	1.00789	−0.06921	0.0910*	
H2B	0.89269	0.99862	0.07747	0.0833*	
H4B	0.51235	0.80448	0.04761	0.0734*	
H7B	0.28306	0.71125	−0.09897	0.0861*	
H8B	0.11890	0.67795	−0.23384	0.1009*	
H9B	0.17607	0.75097	−0.35428	0.0997*	
H10B	0.40379	0.85356	−0.34270	0.0944*	
H15B	0.94904	0.92260	0.20120	0.0821*	
H16C	0.74254	1.02441	−0.22340	0.0911*	
H16D	0.63074	0.96791	−0.30229	0.0911*	
H17D	0.94256	0.90199	−0.20260	0.1391*	
H17E	0.90551	0.93350	−0.29948	0.1391*	
H17F	0.82695	0.83581	−0.27327	0.1391*	
H32B	0.61443	0.89588	0.30842	0.0811*	
H34B	0.90132	0.87866	0.54867	0.0927*	
H35B	1.14430	0.89386	0.49703	0.0978*	
H36B	1.11889	0.90875	0.35045	0.0896*	

### Atomic displacement parameters (Å^2^)

**Table:**

	*U*^11^	*U*^22^	*U*^33^	*U*^12^	*U*^13^	*U*^23^
O1A	0.083 (4)	0.151 (6)	0.090 (4)	0.013 (4)	0.037 (3)	0.013 (4)
O2A	0.117 (5)	0.254 (11)	0.058 (3)	0.014 (6)	0.039 (3)	0.011 (5)
N3A	0.088 (4)	0.101 (5)	0.059 (4)	0.005 (4)	0.023 (3)	0.001 (3)
N12A	0.081 (4)	0.075 (4)	0.052 (3)	0.007 (3)	0.026 (3)	−0.001 (3)
N14A	0.068 (3)	0.089 (4)	0.052 (3)	0.003 (3)	0.020 (3)	0.003 (3)
C1A	0.077 (4)	0.073 (4)	0.071 (5)	0.014 (4)	0.030 (3)	0.004 (4)
C2A	0.067 (4)	0.067 (4)	0.071 (4)	0.012 (3)	0.018 (3)	0.005 (3)
C3A	0.065 (4)	0.077 (4)	0.060 (4)	−0.006 (4)	0.025 (3)	−0.003 (4)
C4A	0.061 (3)	0.076 (4)	0.061 (4)	0.001 (3)	0.024 (3)	−0.002 (3)
C5A	0.054 (3)	0.071 (4)	0.055 (4)	−0.005 (3)	0.019 (3)	−0.003 (3)
C6A	0.053 (3)	0.071 (4)	0.062 (4)	−0.009 (3)	0.019 (3)	0.001 (3)
C7A	0.068 (4)	0.088 (5)	0.066 (4)	0.003 (4)	0.019 (3)	0.007 (4)
C8A	0.071 (4)	0.092 (5)	0.073 (5)	0.006 (4)	0.017 (3)	0.009 (4)
C9A	0.072 (4)	0.095 (6)	0.070 (5)	−0.002 (4)	0.015 (4)	0.018 (4)
C10A	0.080 (4)	0.076 (5)	0.051 (4)	−0.005 (4)	0.020 (3)	0.003 (3)
C11A	0.072 (4)	0.072 (4)	0.062 (4)	−0.015 (4)	0.026 (3)	0.000 (3)
C13A	0.063 (4)	0.064 (4)	0.065 (4)	−0.003 (3)	0.021 (3)	−0.003 (3)
C15A	0.060 (4)	0.084 (5)	0.066 (4)	−0.002 (4)	0.023 (3)	0.002 (4)
C16A	0.087 (5)	0.081 (5)	0.070 (5)	0.014 (4)	0.033 (4)	−0.008 (4)
C17A	0.090 (5)	0.100 (6)	0.079 (5)	0.009 (5)	0.041 (4)	−0.003 (5)
C31A	0.066 (4)	0.073 (4)	0.060 (4)	0.000 (4)	0.020 (3)	0.003 (3)
C32A	0.065 (4)	0.076 (4)	0.055 (4)	0.000 (4)	0.009 (3)	0.004 (3)
C33A	0.071 (4)	0.077 (4)	0.066 (4)	0.009 (4)	0.025 (3)	0.005 (4)
C34A	0.097 (5)	0.099 (6)	0.051 (4)	0.007 (5)	0.015 (4)	0.004 (4)
C35A	0.078 (4)	0.102 (6)	0.064 (4)	0.004 (5)	0.005 (3)	0.004 (4)
C36A	0.067 (4)	0.086 (5)	0.071 (4)	−0.002 (4)	0.017 (3)	0.006 (4)
O1B	0.071 (3)	0.120 (5)	0.088 (4)	−0.006 (3)	0.022 (3)	−0.009 (4)
O2B	0.120 (5)	0.140 (6)	0.066 (4)	−0.005 (4)	0.043 (3)	−0.001 (3)
N3B	0.086 (4)	0.088 (4)	0.069 (4)	−0.010 (4)	0.030 (3)	−0.006 (3)
N12B	0.076 (3)	0.079 (4)	0.055 (3)	−0.005 (3)	0.026 (3)	0.000 (3)
N14B	0.065 (3)	0.076 (4)	0.064 (4)	−0.002 (3)	0.020 (3)	0.001 (3)
C1B	0.080 (4)	0.081 (5)	0.070 (4)	−0.014 (4)	0.024 (4)	0.002 (4)
C2B	0.070 (4)	0.084 (5)	0.054 (4)	−0.011 (4)	0.014 (3)	−0.005 (3)
C3B	0.056 (3)	0.076 (4)	0.057 (4)	0.003 (3)	0.015 (3)	−0.002 (3)
C4B	0.060 (3)	0.066 (4)	0.057 (4)	0.000 (3)	0.012 (3)	0.001 (3)
C5B	0.065 (4)	0.057 (3)	0.059 (4)	−0.005 (3)	0.018 (3)	−0.001 (3)
C6B	0.062 (4)	0.083 (5)	0.056 (4)	0.006 (3)	0.023 (3)	−0.005 (3)
C7B	0.070 (4)	0.087 (5)	0.061 (4)	−0.006 (4)	0.021 (3)	−0.007 (4)
C8B	0.068 (4)	0.104 (6)	0.083 (5)	−0.007 (4)	0.024 (4)	−0.011 (5)
C9B	0.074 (4)	0.115 (7)	0.060 (4)	0.005 (5)	0.015 (3)	−0.010 (4)
C10B	0.077 (4)	0.107 (6)	0.053 (4)	0.006 (4)	0.019 (3)	−0.008 (4)
C11B	0.069 (4)	0.071 (4)	0.051 (3)	0.006 (3)	0.019 (3)	−0.003 (3)
C13B	0.072 (4)	0.071 (4)	0.055 (4)	0.005 (3)	0.020 (3)	0.002 (3)
C15B	0.066 (4)	0.084 (5)	0.057 (4)	−0.001 (4)	0.018 (3)	−0.003 (3)
C16B	0.098 (5)	0.075 (4)	0.060 (4)	−0.003 (4)	0.029 (4)	0.005 (3)
C17B	0.102 (6)	0.105 (6)	0.084 (6)	−0.008 (5)	0.048 (5)	0.003 (5)
C31B	0.063 (4)	0.076 (4)	0.061 (4)	−0.001 (4)	0.014 (3)	−0.004 (4)
C32B	0.071 (4)	0.075 (4)	0.056 (4)	0.004 (4)	0.013 (3)	−0.004 (3)
C33B	0.069 (4)	0.075 (4)	0.062 (4)	−0.001 (4)	0.016 (3)	−0.005 (4)
C34B	0.079 (5)	0.092 (5)	0.058 (4)	−0.005 (4)	0.012 (3)	−0.004 (4)
C35B	0.071 (4)	0.103 (6)	0.064 (4)	−0.002 (4)	0.002 (3)	−0.010 (4)
C36B	0.068 (4)	0.090 (5)	0.068 (4)	−0.007 (4)	0.019 (3)	−0.007 (4)

### Geometric parameters (Å, °)

**Table:**

O1A—N3A	1.190 (10)	C16A—H16B	0.9700
O2A—N3A	1.207 (9)	C17A—H17B	0.9600
O1B—N3B	1.210 (9)	C17A—H17C	0.9600
O2B—N3B	1.216 (9)	C17A—H17A	0.9600
N3A—C33A	1.477 (11)	C32A—H32A	0.9300
N12A—C13A	1.373 (8)	C34A—H34A	0.9300
N12A—C11A	1.388 (10)	C35A—H35A	0.9300
N12A—C16A	1.466 (11)	C36A—H36A	0.9300
N14A—C15A	1.255 (9)	C1B—C2B	1.376 (10)
N14A—C3A	1.427 (8)	C1B—C13B	1.392 (11)
N3B—C33B	1.479 (10)	C2B—C3B	1.407 (11)
N12B—C11B	1.383 (9)	C3B—C4B	1.361 (10)
N12B—C13B	1.395 (8)	C4B—C5B	1.401 (9)
N12B—C16B	1.468 (10)	C5B—C6B	1.450 (9)
N14B—C3B	1.422 (9)	C5B—C13B	1.409 (10)
N14B—C15B	1.254 (9)	C6B—C7B	1.381 (11)
C1A—C13A	1.376 (10)	C6B—C11B	1.424 (9)
C1A—C2A	1.365 (10)	C7B—C8B	1.369 (11)
C2A—C3A	1.412 (11)	C8B—C9B	1.399 (12)
C3A—C4A	1.395 (10)	C9B—C10B	1.357 (12)
C4A—C5A	1.382 (9)	C10B—C11B	1.392 (9)
C5A—C6A	1.443 (9)	C15B—C31B	1.468 (10)
C5A—C13A	1.409 (9)	C16B—C17B	1.497 (13)
C6A—C7A	1.368 (11)	C31B—C32B	1.391 (11)
C6A—C11A	1.427 (10)	C31B—C36B	1.393 (11)
C7A—C8A	1.389 (11)	C32B—C33B	1.375 (9)
C8A—C9A	1.384 (12)	C33B—C34B	1.369 (11)
C9A—C10A	1.380 (12)	C34B—C35B	1.388 (12)
C10A—C11A	1.399 (10)	C35B—C36B	1.393 (11)
C15A—C31A	1.482 (9)	C1B—H1B	0.9300
C16A—C17A	1.492 (13)	C2B—H2B	0.9300
C31A—C36A	1.385 (10)	C4B—H4B	0.9300
C31A—C32A	1.382 (10)	C7B—H7B	0.9300
C32A—C33A	1.365 (10)	C8B—H8B	0.9300
C33A—C34A	1.376 (12)	C9B—H9B	0.9300
C34A—C35A	1.379 (12)	C10B—H10B	0.9300
C35A—C36A	1.361 (11)	C15B—H15B	0.9300
C1A—H1A	0.9300	C16B—H16C	0.9700
C2A—H2A	0.9300	C16B—H16D	0.9700
C4A—H4A	0.9300	C17B—H17D	0.9600
C7A—H7A	0.9300	C17B—H17E	0.9600
C8A—H8A	0.9300	C17B—H17F	0.9600
C9A—H9A	0.9300	C32B—H32B	0.9300
C10A—H10A	0.9300	C34B—H34B	0.9300
C15A—H15A	0.9300	C35B—H35B	0.9300
C16A—H16A	0.9700	C36B—H36B	0.9300
			
O1A—N3A—O2A	122.9 (8)	C33A—C32A—H32A	120.00
O1A—N3A—C33A	119.2 (6)	C33A—C34A—H34A	121.00
O2A—N3A—C33A	117.9 (7)	C35A—C34A—H34A	121.00
C11A—N12A—C13A	108.4 (6)	C34A—C35A—H35A	120.00
C11A—N12A—C16A	125.1 (6)	C36A—C35A—H35A	120.00
C13A—N12A—C16A	126.0 (6)	C31A—C36A—H36A	120.00
C3A—N14A—C15A	119.8 (6)	C35A—C36A—H36A	120.00
O1B—N3B—C33B	118.6 (6)	C2B—C1B—C13B	117.4 (7)
O2B—N3B—C33B	117.8 (7)	C1B—C2B—C3B	122.0 (7)
O1B—N3B—O2B	123.5 (8)	N14B—C3B—C2B	122.7 (6)
C11B—N12B—C13B	107.9 (6)	N14B—C3B—C4B	117.0 (6)
C13B—N12B—C16B	124.7 (6)	C2B—C3B—C4B	120.2 (6)
C11B—N12B—C16B	127.3 (5)	C3B—C4B—C5B	119.6 (6)
C3B—N14B—C15B	121.3 (6)	C4B—C5B—C6B	133.9 (6)
C2A—C1A—C13A	119.2 (7)	C4B—C5B—C13B	119.2 (6)
C1A—C2A—C3A	121.6 (7)	C6B—C5B—C13B	106.8 (6)
N14A—C3A—C2A	124.1 (7)	C5B—C6B—C7B	135.1 (6)
N14A—C3A—C4A	117.3 (6)	C5B—C6B—C11B	105.8 (6)
C2A—C3A—C4A	118.6 (6)	C7B—C6B—C11B	119.0 (6)
C3A—C4A—C5A	120.3 (6)	C6B—C7B—C8B	119.5 (7)
C4A—C5A—C6A	133.4 (6)	C7B—C8B—C9B	121.0 (8)
C4A—C5A—C13A	119.4 (6)	C8B—C9B—C10B	121.1 (7)
C6A—C5A—C13A	107.1 (5)	C9B—C10B—C11B	118.6 (7)
C7A—C6A—C11A	118.9 (6)	N12B—C11B—C6B	109.8 (5)
C5A—C6A—C11A	105.5 (6)	N12B—C11B—C10B	129.5 (6)
C5A—C6A—C7A	135.6 (6)	C6B—C11B—C10B	120.7 (7)
C6A—C7A—C8A	120.8 (7)	N12B—C13B—C1B	128.9 (7)
C7A—C8A—C9A	119.8 (8)	N12B—C13B—C5B	109.5 (6)
C8A—C9A—C10A	121.7 (7)	C1B—C13B—C5B	121.6 (6)
C9A—C10A—C11A	118.2 (7)	N14B—C15B—C31B	122.0 (7)
N12A—C11A—C6A	109.4 (6)	N12B—C16B—C17B	112.4 (7)
N12A—C11A—C10A	130.0 (7)	C15B—C31B—C32B	121.5 (7)
C6A—C11A—C10A	120.6 (7)	C15B—C31B—C36B	120.3 (7)
N12A—C13A—C1A	129.4 (6)	C32B—C31B—C36B	118.1 (7)
N12A—C13A—C5A	109.6 (6)	C31B—C32B—C33B	119.5 (7)
C1A—C13A—C5A	121.0 (6)	N3B—C33B—C32B	118.4 (6)
N14A—C15A—C31A	121.4 (6)	N3B—C33B—C34B	118.8 (6)
N12A—C16A—C17A	111.8 (7)	C32B—C33B—C34B	122.8 (7)
C15A—C31A—C36A	119.9 (6)	C33B—C34B—C35B	118.7 (7)
C15A—C31A—C32A	121.1 (6)	C34B—C35B—C36B	119.2 (7)
C32A—C31A—C36A	119.1 (6)	C31B—C36B—C35B	121.6 (7)
C31A—C32A—C33A	119.2 (7)	C2B—C1B—H1B	121.00
C32A—C33A—C34A	122.0 (7)	C13B—C1B—H1B	121.00
N3A—C33A—C32A	118.7 (7)	C1B—C2B—H2B	119.00
N3A—C33A—C34A	119.2 (7)	C3B—C2B—H2B	119.00
C33A—C34A—C35A	118.4 (7)	C3B—C4B—H4B	120.00
C34A—C35A—C36A	120.4 (7)	C5B—C4B—H4B	120.00
C31A—C36A—C35A	120.9 (7)	C6B—C7B—H7B	120.00
C2A—C1A—H1A	120.00	C8B—C7B—H7B	120.00
C13A—C1A—H1A	120.00	C7B—C8B—H8B	120.00
C1A—C2A—H2A	119.00	C9B—C8B—H8B	119.00
C3A—C2A—H2A	119.00	C8B—C9B—H9B	119.00
C5A—C4A—H4A	120.00	C10B—C9B—H9B	119.00
C3A—C4A—H4A	120.00	C9B—C10B—H10B	121.00
C6A—C7A—H7A	120.00	C11B—C10B—H10B	121.00
C8A—C7A—H7A	120.00	N14B—C15B—H15B	119.00
C9A—C8A—H8A	120.00	C31B—C15B—H15B	119.00
C7A—C8A—H8A	120.00	N12B—C16B—H16C	109.00
C8A—C9A—H9A	119.00	N12B—C16B—H16D	109.00
C10A—C9A—H9A	119.00	C17B—C16B—H16C	109.00
C9A—C10A—H10A	121.00	C17B—C16B—H16D	109.00
C11A—C10A—H10A	121.00	H16C—C16B—H16D	108.00
N14A—C15A—H15A	119.00	C16B—C17B—H17D	110.00
C31A—C15A—H15A	119.00	C16B—C17B—H17E	109.00
N12A—C16A—H16A	109.00	C16B—C17B—H17F	109.00
N12A—C16A—H16B	109.00	H17D—C17B—H17E	109.00
C17A—C16A—H16A	109.00	H17D—C17B—H17F	109.00
C17A—C16A—H16B	109.00	H17E—C17B—H17F	109.00
H16A—C16A—H16B	108.00	C31B—C32B—H32B	120.00
H17B—C17A—H17C	109.00	C33B—C32B—H32B	120.00
C16A—C17A—H17A	110.00	C33B—C34B—H34B	121.00
C16A—C17A—H17B	109.00	C35B—C34B—H34B	121.00
C16A—C17A—H17C	110.00	C34B—C35B—H35B	120.00
H17A—C17A—H17B	109.00	C36B—C35B—H35B	120.00
H17A—C17A—H17C	109.00	C31B—C36B—H36B	119.00
C31A—C32A—H32A	120.00	C35B—C36B—H36B	119.00
			
O1A—N3A—C33A—C32A	−7.9 (13)	C6A—C7A—C8A—C9A	−2.2 (13)
O1A—N3A—C33A—C34A	174.5 (9)	C7A—C8A—C9A—C10A	−0.3 (14)
O2A—N3A—C33A—C32A	169.9 (10)	C8A—C9A—C10A—C11A	1.6 (13)
O2A—N3A—C33A—C34A	−7.8 (13)	C9A—C10A—C11A—N12A	175.9 (8)
C13A—N12A—C11A—C6A	−0.6 (8)	C9A—C10A—C11A—C6A	−0.4 (12)
C13A—N12A—C11A—C10A	−177.3 (8)	N14A—C15A—C31A—C36A	−172.7 (8)
C16A—N12A—C11A—C6A	−173.6 (7)	N14A—C15A—C31A—C32A	6.0 (12)
C16A—N12A—C11A—C10A	9.8 (13)	C15A—C31A—C36A—C35A	179.5 (9)
C11A—N12A—C13A—C1A	178.7 (8)	C15A—C31A—C32A—C33A	179.4 (8)
C11A—N12A—C13A—C5A	1.2 (8)	C36A—C31A—C32A—C33A	−1.9 (12)
C16A—N12A—C13A—C1A	−8.5 (13)	C32A—C31A—C36A—C35A	0.8 (14)
C16A—N12A—C13A—C5A	174.0 (7)	C31A—C32A—C33A—C34A	0.6 (13)
C11A—N12A—C16A—C17A	91.1 (9)	C31A—C32A—C33A—N3A	−177.0 (7)
C13A—N12A—C16A—C17A	−80.7 (10)	N3A—C33A—C34A—C35A	179.4 (8)
C15A—N14A—C3A—C2A	32.0 (12)	C32A—C33A—C34A—C35A	1.8 (14)
C15A—N14A—C3A—C4A	−151.4 (8)	C33A—C34A—C35A—C36A	−2.9 (15)
C3A—N14A—C15A—C31A	−178.2 (7)	C34A—C35A—C36A—C31A	1.6 (15)
O1B—N3B—C33B—C34B	−179.3 (8)	C13B—C1B—C2B—C3B	1.2 (12)
O2B—N3B—C33B—C32B	−179.1 (8)	C2B—C1B—C13B—N12B	175.9 (8)
O1B—N3B—C33B—C32B	−0.5 (11)	C2B—C1B—C13B—C5B	−1.4 (12)
O2B—N3B—C33B—C34B	2.1 (12)	C1B—C2B—C3B—N14B	−176.7 (8)
C16B—N12B—C11B—C6B	173.7 (7)	C1B—C2B—C3B—C4B	−1.1 (12)
C16B—N12B—C11B—C10B	−7.8 (13)	N14B—C3B—C4B—C5B	177.0 (6)
C11B—N12B—C16B—C17B	−96.0 (9)	C2B—C3B—C4B—C5B	1.1 (11)
C13B—N12B—C16B—C17B	79.7 (9)	C3B—C4B—C5B—C6B	−176.4 (8)
C16B—N12B—C13B—C1B	7.6 (13)	C3B—C4B—C5B—C13B	−1.3 (10)
C11B—N12B—C13B—C1B	−176.0 (8)	C4B—C5B—C6B—C7B	−3.7 (15)
C11B—N12B—C13B—C5B	1.6 (8)	C4B—C5B—C6B—C11B	174.1 (7)
C13B—N12B—C11B—C10B	176.0 (8)	C13B—C5B—C6B—C7B	−179.3 (9)
C16B—N12B—C13B—C5B	−174.8 (7)	C13B—C5B—C6B—C11B	−1.5 (8)
C13B—N12B—C11B—C6B	−2.6 (8)	C4B—C5B—C13B—N12B	−176.4 (6)
C15B—N14B—C3B—C2B	−31.6 (12)	C4B—C5B—C13B—C1B	1.4 (11)
C3B—N14B—C15B—C31B	−179.6 (7)	C6B—C5B—C13B—N12B	0.0 (8)
C15B—N14B—C3B—C4B	152.6 (8)	C6B—C5B—C13B—C1B	177.8 (7)
C2A—C1A—C13A—N12A	−176.3 (7)	C5B—C6B—C7B—C8B	175.2 (9)
C13A—C1A—C2A—C3A	0.3 (12)	C11B—C6B—C7B—C8B	−2.3 (12)
C2A—C1A—C13A—C5A	0.9 (11)	C5B—C6B—C11B—N12B	2.5 (8)
C1A—C2A—C3A—C4A	−0.4 (12)	C5B—C6B—C11B—C10B	−176.2 (7)
C1A—C2A—C3A—N14A	176.2 (7)	C7B—C6B—C11B—N12B	−179.3 (7)
N14A—C3A—C4A—C5A	−177.6 (7)	C7B—C6B—C11B—C10B	2.0 (11)
C2A—C3A—C4A—C5A	−0.7 (11)	C6B—C7B—C8B—C9B	0.7 (14)
C3A—C4A—C5A—C13A	1.9 (11)	C7B—C8B—C9B—C10B	1.4 (15)
C3A—C4A—C5A—C6A	177.9 (7)	C8B—C9B—C10B—C11B	−1.7 (14)
C4A—C5A—C6A—C11A	−175.5 (8)	C9B—C10B—C11B—N12B	−178.4 (8)
C13A—C5A—C6A—C7A	179.5 (9)	C9B—C10B—C11B—C6B	0.0 (12)
C13A—C5A—C6A—C11A	0.8 (8)	N14B—C15B—C31B—C32B	−7.7 (13)
C4A—C5A—C13A—N12A	175.7 (6)	N14B—C15B—C31B—C36B	168.9 (8)
C4A—C5A—C13A—C1A	−2.1 (10)	C15B—C31B—C32B—C33B	177.8 (8)
C6A—C5A—C13A—N12A	−1.3 (8)	C36B—C31B—C32B—C33B	1.2 (12)
C6A—C5A—C13A—C1A	−179.0 (7)	C15B—C31B—C36B—C35B	−176.8 (9)
C4A—C5A—C6A—C7A	3.1 (15)	C32B—C31B—C36B—C35B	−0.1 (13)
C11A—C6A—C7A—C8A	3.3 (12)	C31B—C32B—C33B—N3B	178.8 (7)
C5A—C6A—C11A—N12A	−0.2 (8)	C31B—C32B—C33B—C34B	−2.4 (13)
C5A—C6A—C11A—C10A	176.9 (7)	N3B—C33B—C34B—C35B	−178.8 (8)
C7A—C6A—C11A—N12A	−179.1 (7)	C32B—C33B—C34B—C35B	2.5 (14)
C7A—C6A—C11A—C10A	−2.1 (11)	C33B—C34B—C35B—C36B	−1.3 (15)
C5A—C6A—C7A—C8A	−175.2 (8)	C34B—C35B—C36B—C31B	0.2 (15)
